# Efficient and accurate whole genome assembly and methylome profiling of *E. coli*

**DOI:** 10.1186/1471-2164-14-675

**Published:** 2013-10-03

**Authors:** Jason G Powers, Victor J Weigman, Jenny Shu, John M Pufky, Donald Cox, Patrick Hurban

**Affiliations:** Expression Analysis, A Quintiles Company, Durham, NC 27713 USA

**Keywords:** Genome assembly, Illumina MiSeq, Ion Torrent PGM, PacBio RS, Base modifications, *E. coli*, Hybrid assembly, 5mC

## Abstract

**Background:**

With the price of next generation sequencing steadily decreasing, bacterial genome assembly is now accessible to a wide range of researchers. It is therefore necessary to understand the best methods for generating a genome assembly, specifically, which combination of sequencing and bioinformatics strategies result in the most accurate assemblies. Here, we sequence three *E. coli* strains on the Illumina MiSeq, Life Technologies Ion Torrent PGM, and Pacific Biosciences RS. We then perform genome assemblies on all three datasets alone or in combination to determine the best methods for the assembly of bacterial genomes.

**Results:**

Three *E. coli* strains – BL21(DE3), Bal225, and DH5α – were sequenced to a depth of 100× on the MiSeq and Ion Torrent machines and to at least 125× on the PacBio RS. Four assembly methods were examined and compared. The previously published BL21(DE3) genome [GenBank:AM946981.2], allowed us to evaluate the accuracy of each of the BL21(DE3) assemblies. BL21(DE3) PacBio-only assemblies resulted in a 90% reduction in contigs versus short read only assemblies, while N50 numbers increased by over 7-fold. Strikingly, the number of SNPs in PacBio-only assemblies were less than half that seen with short read assemblies (~20 SNPs vs. ~50 SNPs) and indels also saw dramatic reductions (~2 indel >5 bp in PacBio-only assemblies vs. ~12 for short-read only assemblies). Assemblies that used a mixture of PacBio and short read data generally fell in between these two extremes. Use of PacBio sequencing reads also allowed us to call covalent base modifications for the three strains. Each of the strains used here had a known covalent base modification genotype, which was confirmed by PacBio sequencing.

**Conclusion:**

Using data generated solely from the Pacific Biosciences RS, we were able to generate the most complete and accurate *de novo* assemblies of *E. coli* strains*.* We found that the addition of other sequencing technology data offered no improvements over use of PacBio data alone. In addition, the sequencing data from the PacBio RS allowed for sensitive and specific calling of covalent base modifications.

**Electronic supplementary material:**

The online version of this article (doi:10.1186/1471-2164-14-675) contains supplementary material, which is available to authorized users.

## Background

Bacteria make up an enormous portion of the world around us. Some estimate that there are 4–6 × 10^30^ prokaryotes on Earth today [1]. They are known to live in virtually every environment on Earth, playing critical roles in both human health (e.g. digestion and disease) and the global ecosystem (e.g. decomposition, oxygen production). Understanding their genetic diversity is a crucial part in understanding how bacteria have evolved to play these various roles. Recent advances in sequencing have made these studies more accessible than ever, with large amounts of sequencing data readily generated from a variety of machines at both a reasonable price and with turnaround times measured in days, not weeks. Increasingly, this accessibility is being applied to both research and clinical studies and is revolutionizing our understanding of bacterial genetics and their diversity. These rapid gains in understanding of bacterial genetics promises to exponentially expand our understanding of the interplay between bacteria, the environment, and human health.

Before such promises can be fulfilled it is important to develop best practices for how the genetic profiles of microbes are studied. Which machines are used to sequence the bacteria, the reagents used to prepare the DNA for sequencing, and the software used to analyze the resulting data all influence the depth of knowledge one can gain from any given sequencing experiment. An important step in understanding the genetics of bacteria, or indeed any organism, is to understand the genome – its structure, size and gene organization. Assembling these genomes from next generation sequencing data is thus a critical task towards fully understanding bacterial functions and phylogenetic relationships – an assembly that is highly fractured or incorrect can impede further studies of gene expression, function, and phylogenetic relationships, while accurate assemblies open the door to a wealth of further studies.

**Figure 1:**
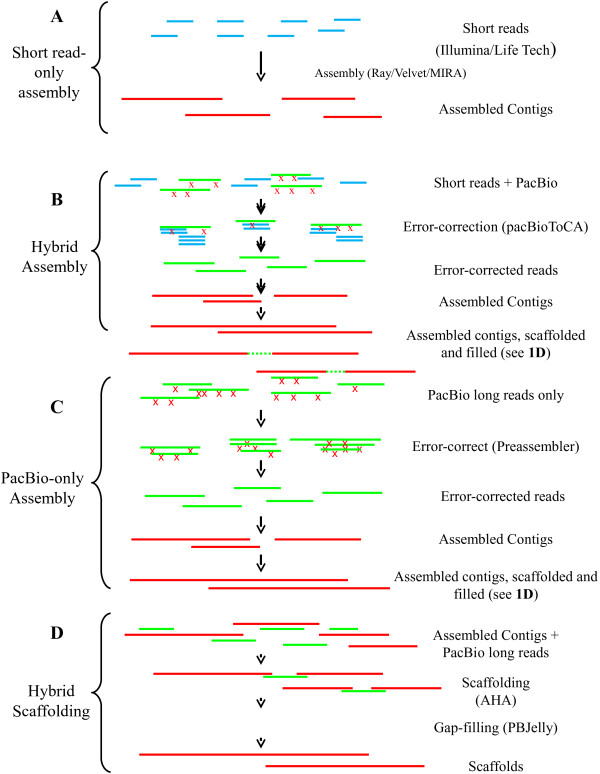
**The four bacterial assembly strategies examined here. A**. Short read only assembly. Short next generation sequencing reads generated by the Illumina or Life Technologies platforms (in blue) are assembled with any number of assembly software packages into contiguous sequences (contigs; in red). Here, the software packages Velvet, Ray, and MIRA were evaluated. **B**. Hybrid Assembly. In this method of assembly, short, higher accuracy reads from the Illumina/Life Technologies platforms (blue), error correct the long, lower accuracy PacBio reads (in green with red Xs denoting basecalling errors) via alignment. The software package pacBioToCA was used for this purpose. These error-corrected reads are then assembled into contigs (in red) using a software package suited to long read assembly such as the Celera assembler. Assembled contigs can be further scaffolded and gap-filled as in **D**. **C**. In PacBio-only assembly, long reads are aligned to each other, enabling self-correction. These self-corrected reads are assembled into contigs and can be further scaffolded and gap-filled as in **D**. **D**. Hybrid scaffolding, in this method, preassembled contigs (red), derived from any of the methods laid out in **A-C**, are scaffolded with long PacBio reads (in green). These long scaffolds, which can contain long strings of “N”s (dotted green lines) in between the contigs, are then gap-filled with the program PBJelly.

Two benchtop sequencers dominate the market today, the Ion Torrent PGM from Life Technologies [2] and the MiSeq from Illumina [3]. Both facilitate rapid, cost-effective sequencing. Data generated from these machines can be paired-end or single-end, and can range from 150 nt to ~400 nt in length. Assembling a genome of several million bases or more with reads of this length is a complicated process, although there are a number of freely-available tools to assemble the genome of bacteria using data generated from these machines [4]. Although read lengths continue to improve on these instruments, we will refer to this method of assembly using the historical nomenclature of “short read only assembly” (Figure 1A) [5].

Just as the Ion Torrent and MiSeq can sequence a bacterial genome in less than a day, so can a third sequencing platform, the Pacific Biosciences RS (PacBio). In addition to fast sequencing reactions, the PacBio distinguishes itself from the other two machines in at least three ways. First, the PacBio produces very long reads, some as long as 20 kb, with average read lengths of 4-5 kb, making the single, continuous reads generated by this machine an order of magnitude longer than any other [6]. Second, the library preparation process does not include an amplification step, meaning DNA is sequenced in its native form, a single molecule at a time. This style of sequencing in turn allows for the third distinguishing characteristic of the PacBio – the ability to call covalent base modifications [7]. While understanding the nucleotide sequence of bacterial genomes is an important step in understanding the biology of any given bacterial species, knowledge of the epigenome will also prove to be useful in elucidating biological functions, and indeed has already been applied to pathogenic bacteria [8, 9]. Recent work has also defined a roadmap for extending base modification identification to RNA samples, using the HIV-1 reverse transcriptase enzyme instead of a DNA polymerase, providing another sequencing application unique to the PacBio [10, 11].

The long read lengths generated by PacBio sequencing come at a price: the average base quality of any given base is substantially lower than that of either the Illumina or Life Technology machines. Still, the long read lengths offer a significant advantage during assembly, and this has led at least two groups to design algorithms that correct the errors found in long PacBio reads using the shorter, yet higher accuracy reads from the Illumina and Life Technology machines [12, 13]. Referred to in this paper as Hybrid Assembly, these error corrected reads are more amenable to traditional assembly using overlap graph assemblers like the Celera software package (Figure 1B) [14]. More recently, both PacBio and the research group responsible for Celera have released versions of their software packages which self-corrects PacBio reads by aligning reads to each other and generating consensus sequences [15–17]. These self-corrected, PacBio-only reads can be fed into the same assemblers as the hybrid reads (Figure 1C).

Bacterial genome sequencing and assembly is not a new phenomenon, and indeed many researchers have sequenced bacterial genomes and generated draft assemblies [18]. It is therefore not uncommon for researchers to look to improve upon their pre-existing assemblies through a process referred to here as Hybrid Scaffolding (Figure 1D). In this method, a pre-existing bacterial assembly is improved by using PacBio long reads to connect two pre-assembled contigs into a longer sequence called a scaffold. These scaffolds consist of the previously assembled contigs connected by a string of N’s in between, called gaps. Many of these intervening gaps can subsequently be filled using PBJelly software, ultimately resulting in a more complete assembly [19].

In this report, we address best practices for bacterial assembly by sequencing the well characterized *E. coli* strain BL21(DE3) [20, 21] on the Illumina MiSeq, Ion Torrent, and PacBio, performing *de novo* assemblies under a variety of conditions, and comparing those assemblies to the published genome. We examine the four primary methods of bacterial assembly – short read only assemblies, hybrid scaffolding, hybrid assembly, and PacBio-only assembly. We then extend those results to sequence and assemble two other *E. coli* strains, Bal225 and the common laboratory strain DH5α [22].

Finally, with assembled bacterial strains in hand, we use PacBio sequencing to examine covalent base modifications present in each of the bacterial strains. The three predominant methyltransferases in E. coli are the DAM methylase, the DCM methylase, and the EcoKI methylase. Each methylase has a specific base substrate within a larger sequence motif which it targets for methylation. The DAM methylase targets the N6 position of the adenine in the sequence motif GA*TC. The DCM methylase specifically methylates the second cytosine in the sequence motifs CC*AGG and CC*TGG. Finally, the EcoKI methylase modifies the second or third adenine in the sequence motifs AA*C(N6)GTGC and GCA*C(N6)GTT, respectively[23–28]. The three E. coli strains in this study were chosen specifically to address the accuracy and specificity of the PacBio sequence data and associated software to call these modifications on real world data. Whereas DH5α has all three enzymes, BL21(DE3) is deficient in EcoKI and DCM, while Bal225 lacks the DAM and DCM methylases.

## Results

### Short read assembly

We began our assembly comparisons with BL21(DE3), since the completed genome is available from NCBI, and thus provides a reference against which we can compare the various *de novo* assemblies [GenBank:AM946981.2, 21]. BL21(DE3) was sequenced to a depth of over 100× on both the MiSeq and Ion Torrent PGM machines. MiSeq reads were paired end, 150 bp in length, while the Ion Torrent reads were single-end, approximately 200 bp in length on average (Additional file 1: Table S1).

Several short read assembly software packages are available, and we chose to examine three: Velvet, Ray, and MIRA [29–31]. Ray and Velvet have both been extensively used and validated in competitions such as Assemblathon 1 and 2, while MIRA has been widely used for over a decade [32, 33]. To investigate the effects of assembly at lower coverages, we performed assemblies using coverages of 25×, 50×, 75×, and 100×, by randomly downsampling using the custom script randomFQ [34].

We first assembled data from both benchtop sequencers using Velvet, an extensively used de Bruijn graph assembler [29]. Unfortunately, Velvet’s assembly methodology resulted in very poor performance for Ion Torrent assemblies, and we therefore only report the data for MiSeq assemblies. As a de Bruijn graph assembler, Velvet was originally published using Solexa data, and the poor performance with Ion Torrent data is likely due to its inability to cope with the Ion Torrent error profile. For the MiSeq data we assembled the data using 12 different Kmers, ranging from 21 to 63, for each of the different coverage depths, resulting in 48 MiSeq assemblies with Velvet. Each of these assemblies was examined on the basis of contig number, max contig length, percent of contigs >500, and 21 bp dup-mer percentage. The 21 bp dup-mer statistic is based on the number of unique 21 bp kmers. The statistic, (#21 bp-kmers occurring > 1 time)/(total # of unique 21 bp kmers), can be used to generally evaluate the number of expanded or collapsed repeats, especially when one knows the expected dup-mer number (based on a known reference). A lower than expected number suggests the presence of collapsed repeats, while a higher than expected number indicates expanded repeats. For the reference BL21(DE3) genome, the 21 bp dup-mer is 0.7. Taking all of these statistics under consideration, the most complete Velvet assembly for each of the MiSeq coverages is reported in Table 1.

**Table 1:** **Short read only assembly statistics**

Assembler	Data type	kmer	Approx. coverage	# Contigs	% Contigs > 500	Max contig size	N50	Dup-mer 21	Assembly size	% Assembly size
Velvet	MiSeq	**59**	**100X**	**154**	**54.55%**	**430066**	**119241**	**0.28**	**4493252**	**98.56%**
		59	75X	156	53.21%	413653	119171	0.30	4493894	98.57%
		59	50X	144	55.56%	415116	118491	0.28	4493686	98.57%
		59	25X	244	68.03%	212504	48994	0.26	4494206	98.58%
Ray	MiSeq	**36**	**100X**	**93**	**78.49%**	**332975**	**111161**	**1.67**	**4565040**	**100.13%**
		36	75X	90	85.56%	350117	111227	0.47	4550209	99.81%
		36	50X	100	90.00%	213879	86298	0.45	4495395	98.61%
		36	25X	292	93.15%	79672	26198	0.5	4499709	98.70%
	Ion	29	100X	734	88.56%	34241	10008	2.34	4545446	99.70%
		29	75X	578	87.02%	58878	13621	1.64	4493104	98.56%
		29	50X	440	85.00%	85800	18468	1.01	4499305	98.69%
		**29**	**25X**	**415**	**86.51%**	**75474**	**19997**	**0.36**	**4470372**	**98.06%**
MIRA	MiSeq	n/a	100X	1260	6.51%	388423	115369	2.16	4754899	104.30%
		n/a	75X	457	22.98%	284700	96674	0.85	4630082	101.56%
		**n/a**	**50X**	**321**	**51.09%**	**221854**	**48362**	**1.01**	**4589987**	**100.68%**
		n/a	25X	1071	74.51%	33745	8703	1.31	4545144	99.70%
	Ion	n/a	100X	697	10.33%	493665	180738	2.34	4763496	104.49%
		n/a	75X	429	19.58%	401639	128626	1.60	4674816	102.54%
		n/a	50X	221	37.10%	351325	144583	1.03	4598473	100.87%
		**n/a**	**25X**	**153**	**62.75%**	**281400**	**106822**	**0.73**	**4559337**	**100.01%**

The bolded assemblies represent the best assembly for the specified combination of sequencer and software.

Next we examined Ray, another de Bruijn graph assembler [30]. Both MiSeq and Ion Torrent data were assembled across a variety of kmer sizes (12 per coverage for a total of 96 different assemblies). Each assembly was evaluated using the same metrics used for Velvet to determine the most complete assembly for each coverage/data source type. Unlike Velvet, Ray was able to assemble the Ion Torrent data with reasonable results, however still performed better overall with the MiSeq data, again likely because de Bruijn graph assemblers do poorly with lower quality reads The most complete assemblies for each combination of data type and coverage are reported in Table 1.

Finally, the MIRA assembler was applied to our datasets [31]. MIRA produced the most complete Ion Torrent assembly, with optimal results produced with 25× coverage. In contrast to Velvet and Ray, the MIRA assembler is an overlap graph based assembler and has a specific parameter set tailored to Ion Torrent data, which may account for these superior results. On the other hand, MIRA struggled with the MiSeq data, possibly because these libraries were constructed using the Illumina Nextera kit. Nextera kits utilize transposon-mediated fragmentation, and in our hands the resulting fragments had bimodal insert-size distributions, as opposed to the typical normal distributions that are characteristic of other fragmentation methods, such as acoustic methods (Additional file 2: Figure S1). Again, the most complete× assemblies based on contig number, percent of contigs > 500, max contig size, etc. are reported in Table 1.

**Figure 2:**
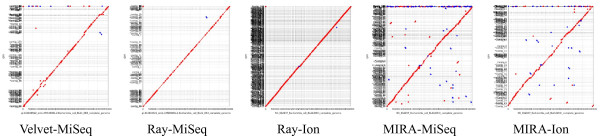
**Dot plots of short read only assemblies.** BL21(DE3) assemblies highlighted in Table 1 were aligned and plotted against the reference genome using MUMmer. The Y-axis shows the various contigs. Deviations from the middle line indicate positions of misassembly. Note the Ray-Ion assembly has the best fitting line but also the largest number of contigs.

After determining the most complete short read assemblies for each combination of assembler/coverage/data type we were left with 20 different assemblies (four Velvet assemblies and eight each for Ray and MIRA). While statistics such as number of contigs, percent of contigs > 500, and max contig size can be used to evaluate assembly completeness, they are poor measures of assembly correctness. To further evaluate the assemblies based on correctness, each of the twenty most complete assemblies were run through an assembly evaluation script utilizing the MUMmer toolkit against the published BL21(DE3) genome sequence [35, 36]. This package generates many statistics about the accuracy of an assembly compared to a reference. A selection of these statistics can be seen in Table 2, and based on these statistics coupled with completeness statistics in Table 1, five different assemblies were chosen as the best for each combination of assembler/data type (highlighted in bold in Tables 1 and 2). To better visualize assembly-correctness, dot plots for these five assemblies were generated (Figure 2). These figures, when combined with the statistics in Tables 1 and 2, demonstrate that the most accurate assemblies were those performed by Ray.

**Table 2:** **Evaluating the short-read only assemblies**

Assembler	Data type	kmer	Approx. coverage	Avg identity	Indels < 5 bp	Indels > = 5 bp	Inversions	Unalignable contigs	Relocations	SNPs
Velvet	MiSeq	**59**	**100X**	**99.99**	**13**	**38**	**2**	**0**	**16**	**42**
		59	75X	99.99	23	31	0	0	12	110
		59	50X	99.99	18	36	1	0	17	88
		59	25X	99.99	36	129	0	0	8	96
Ray	MiSeq	**36**	**100X**	**100**	**1**	**6**	**2**	**19**	**2**	**9**
		36	75X	99.99	5	10	0	11	2	35
		36	50X	99.99	2	3	0	8	2	26
		36	25X	100	5	5	1	4	4	41
	Ion	29	100X	99.86	5645	29	0	4	1	67
		29	75X	99.9	4209	14	0	4	1	58
		29	50X	99.93	2784	7	0	0	0	58
		**29**	**25X**	**99.95**	**1933**	**3**	**0**	**1**	**1**	**54**
MIRA	MiSeq	n/a	100X	99.99	8	6	13	2	15	73
		n/a	75X	99.99	20	4	11	0	11	124
		**n/a**	**50X**	**99.99**	**31**	**5**	**15**	**0**	**9**	**113**
		n/a	25X	99.99	9	7	7	0	7	81
	Ion	n/a	100X	99.98	207	6	11	4	11	50
		n/a	75X	99.98	239	8	14	2	9	42
		n/a	50X	99.98	292	10	9	1	8	58
		**n/a**	**25X**	**99.98**	**524**	**10**	**9**	**0**	**3**	**34**

The bolded assemblies represent the best assembly for the specified combination of sequencer and software.

### Hybrid scaffolding

In addition to the short read data, we sequenced BL21(DE3) to 185X coverage on the Pacific Biosciences RS. Raw PacBio reads averaged ~3.7 kb in length. This dropped to approximately 2 kb in length after adapter removal, which reflects the average library DNA insert size (Additional file 1: Table S1). Each of the five short-read assemblies were improved by connecting the contigs with PacBio long reads. In this two step process, long PacBio reads are first used to connect two distinct contigs. The connected contigs, commonly referred to as scaffolds, are joined with unknown intervening sequences labeled as N’s. These strings of N’s, called gaps, can then be filled in using the PBJelly software package [19]. Using the AHA scaffolder (part of PacBio’s SMRT Analysis software package) [15], contigs were scaffolded together using ~25×, 55×, or 110× PacBio long read coverage, and gap-filled with PBJelly.

In all cases examined, the number of un-connected contigs was reduced by 30% or more. The max sequence length increased by 40% or better, and in some cases the max sequence length was over twice that obtained with the short read only assemblies. For example, adding in 55x coverage of PacBio to the MIRA/MiSeq assembly increased the maximum sequence length by over 3 fold, from 221,854 to 716,302. In two thirds of the cases examined, the resulting scaffolds were less than 100 in number, far better than short read only assemblies alone, for which only 1 in 10 resulted in less than 100 contigs (Table 3). These scaffolds were largely contiguous, as PBJelly was generally able to fill in the gaps between scaffolded contigs. With the exception of the Ray-Ion Torrent assembly, the un-filled gaps numbered 23 or fewer, with median lengths of less than 250 bp. Improvements were seen when incrementally more PacBio long read coverage was added to the scaffolding process, although there were diminishing returns, especially in contig reduction. At least 70% of total gains in the reduction of contigs were achieved with just 25× coverage. Similarly, on average 64% of the maximum sequence length improvements were seen with 25× coverage, and 60% of the N50 gains. These results indicate that at least for these assemblies, 25× PacBio long read coverage was enough to achieve the majority of the gains available using the hybrid scaffolding approach.

**Table 3:** **Hybrid scaffolding assembly statistics**

Original assembler	Original data type	Original coverage	Approx. PacBio coverage	# Scaffolds	% improvement	Max scaffold size	% improvement	N50	% improvement	Total bases	Gaps	Median gap size
Velvet	MiSeq	75X	25X	94	38.96%	789973	183.69%	297566	249.55%	4530346	20	141
			55X	86	44.16%	1184298	275.38%	581705	487.84%	4529781	15	104
			110X	77	50.00%	1107981	257.63%	581889	487.99%	4530783	14	103
Ray	MiSeq	50X	25X	56	39.78%	456422	137.07%	261284	235.05%	4579304	1	25
			55X	47	49.46%	768167	230.70%	297488	267.62%	4574295	0	n/a
			110X	52	44.09%	456422	137.07%	241297	217.07%	4578631	0	n/a
	Ion	25X	25X	92	77.83%	394228	522.34%	186135	930.81%	4605573	307	201
			55X	90	78.31%	324309	429.70%	179686	898.56%	4579067	62	127
			110X	83	80.00%	466975	618.72%	201280	1006.55%	4592864	69	175
MIRA	MiSeq	50X	25X	201	37.38%	661174	298.02%	249860	516.65%	4597589	9	209
			55X	179	44.24%	716302	322.87%	394266	815.24%	4600672	17	110
			110X	176	45.17%	759703	342.43%	433542	896.45%	4606800	23	118
	Ion	25X	25X	112	26.80%	477985	169.86%	141498	132.46%	4569060	7	104
			55X	105	31.37%	477631	169.73%	149865	140.29%	4569565	15	120
			110X	93	39.22%	537668	191.07%	200457	187.66%	4571203	17	230

**Figure 3:**
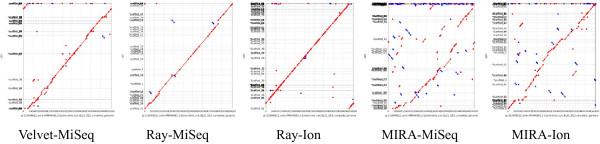
**Dot plots of hybrid scaffolding.** The assemblies highlighted in Table 1 were scaffolded and gap-filled with varying coverages of PacBio reads. The assembled results with ~100× PacBio coverage were aligned to the reference BL21(DE3) and dot plots were generated. Note the more contiguous assemblies, but with more errors as compared to those in Figure 2.

While significant gains were seen in assembly completeness, hybrid scaffolding introduced a significant number of errors into the short read assemblies, especially short indels, a well-known error profile of the PacBio (Table 4). The dot-plots underscored the effect that these new errors had on accuracy, with striking degradation of assembly accuracy in some instances (Figure 3). For example, hybrid scaffolding doubled the number of SNPs and indels greater than 5 bp, and tripled the number of relocations in the MIRA-Ion Torrent assembly. Indels less than 5 bp went up dramatically in many of the assemblies, in the case of the Ray-MiSeq assembly going from 1 to nearly 200.

**Table 4:** **Evaluation of hybrid scaffolding assemblies**

Original assembler	Original data type	Original coverage	Approx. PacBio coverage	Avg identity	Indels < 5 bp	Indels > = 5	Inversions	Missing assembly contigs	Relocation	SNPs
Velvet	MiSeq	75X	25X	99.98	416	36	4	0	31	93
			55X	99.98	471	47	3	0	25	107
			110X	99.98	299	49	2	0	24	88
Ray	MiSeq	50X	25X	99.99	191	10	3	19	3	16
			55X	99.99	210	10	2	19	2	33
			110X	99.99	185	13	3	19	2	33
	Ion	25X	25X	99.95	2092	244	8	1	39	65
			55X	99.93	3000	93	3	1	13	206
			110X	99.93	2732	79	0	1	20	171
MIRA	MiSeq	50X	25X	99.98	199	23	18	0	14	138
			55X	99.98	140	18	19	0	17	103
			110X	99.98	90	17	21	0	19	92
	Ion	25X	25X	99.97	682	14	12	0	9	59
			55X	99.97	650	14	12	0	11	92
			110X	99.97	648	19	13	0	11	74

### Hybrid assembly

A third method of assembly involves using the short, higher accuracy reads generated by Illumina or Life Technologies sequencers to error-correct the long PacBio reads and use those corrected reads for assembly (see Figure 1B). Outside of the Pacific Biosciences analysis package there are at least two pieces of software do this, here we chose pacBioToCA, which is part of the Celera assembler package [12, 13].

The 185x PacBio coverage and a downsampled subset (110×) were error-corrected with either 100× coverage of Ion Torrent data or 100× coverage of MiSeq data. Following this error correction, corrected reads were sorted on length and downsampled to two different coverage targets. Previously published work has demonstrated that the best error-corrected read assemblies result from depths of 12-25× [12]. We therefore downsampled the error-corrected reads targeting two coverages of 12-15× and 20-25×. Statistics for both downsampled data sets for both small read technologies can be found in Additional file 3: Table S2. This left us with four data sets each for the Ion Torrent and MiSeq hybrid correction to assembly 1) the full data-set, error-corrected and downsampled to 12-15×, 2) the full data-set error-corrected, and downsampled to 20-25×, 3) the subset of data, error-corrected and downsampled to 12-15×, and 4) the subset, error-corrected and downsampled to 20-25×. The Ion Torrent error-corrected subsets of data were downsampled by selecting for corrected reads greater than 3000 (for 20-25×) and 4000 (for 12-15×) nucleotides in length, while the MiSeq error-corrected subset of data was downsampled for reads greater than 2500 and 3250, respectively. When more PacBio data was added, the downsampling cutoffs were adjusted upwards, to 4000 and 5000 minimum read lengths for the Ion Torrent corrected data and 3250 and 4250 for the MiSeq corrected data.

Each of the eight different corrected data sets was assembled with Celera using 10 different parameters (see Additional file 4: Table S3 for exact parameters), for a total of 80 different assemblies. The most accurate assembly was chosen based on the software package amosvalidate coupled with FRCurve [37, 38]. Amosvalidate is a software package designed to identify mis-assemblies based on a variety of metrics, each of which may indicate a poor assembly. Importantly, this software does not rely on a reference, instead attempting to identify assembly problems in *de novo* assemblies. These potential mis-assembly features can be plotted via FRCurve, with feature counts along the x-axis and total bases covered on the y-axis (see Additional file 5: Figure S2 for a representative curve). The user can then choose the assembly that covers the most bases with the least number of features. Across all samples we chose the assembly with the least features at 90% coverage of the putative genome size (4.5 Mb). The contigs from the chosen assemblies were then run through the same pipeline as those contigs examined in the Hybrid Scaffolding approach, i.e. scaffolded together with the AHA package and filled in with PBJelly. Assembly statistics can be found in Table 5.

**Table 5:** **Hybrid and PacBio-only assembly statistics**

Correction method	Starting PacBio coverage	Contigs	N50	Mean	Max	Total bases	Scaffolds	N50	Mean	Max	Total bases
Ion	110X	25	549762	188222	793657	4705573	23	694467	204622	1763628	4706308
	185X	31	360777	153272	869306	4751442	27	1231116	176143	1631314	4755875
MiSeq	110X	42	174522	107856	364773	4529989	30	328144	151322	588420	4539669
	185X	49	140368	92321	349122	4523744	36	349122	125872	648829	4531394
Preassembler	110X	22	543086	212632	2026296	4677908	19	696126	246383	2200813	4681278
	185X	21	737479	224642	1239560	4717483	21	737479	224642	1239560	4717483

**Figure 4:**
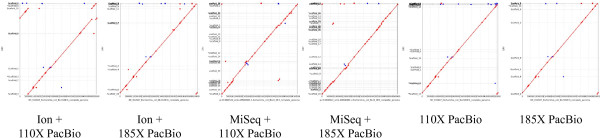
**Dot plots of hybrid and PacBio-only assemblies.** BL21(DE3) was assembled with varying conditions as described. The best assembly for each combination of technology and PacBio coverage was determined using Amosvalidate. Assemblies were scaffolded with AHA, gap-filled with PBJelly and finally polished with Quiver. The resulting assemblies were aligned to the reference genome and dot plots generated with MUMmer.

In terms of assembly accuracy, the MiSeq hybrid assemblies had approximately 10-fold fewer short indels than the Ion Torrent hybrid assemblies. Otherwise, the MiSeq and Ion Torrent hybrid assemblies were comparable in terms of accuracy. No assembly produced a contig or scaffold that was un-alignable to the reference genome, and all had identities that were over 99.9% (Table 6). However, the Ion Torrent hybrid assemblies were more complete than the MiSeq assemblies. When examining the number of contigs, mean, max, and N50 numbers, the Ion Torrent hybrid assemblies produced assemblies that were demonstrably superior to the MiSeq assemblies. For example, with 110× coverage the Ion Torrent hybrid assembly resulted in a maximum contig length of 793,657, more than twice the maximum seen when the MiSeq was used (364,773). These results can be traced back to the error correction performed with each set of data. In each of the samplings, reads error-corrected with the Ion Torrent ultimately had longer lengths than those error-corrected with the MiSeq data. These longer reads in turn resulted in more complete assemblies. Dot plots were generated with the assembled data showing that both hybrid assemblies were highly accurate and largely complete (Figure 4).

**Table 6:** **Evaluating Hybrid and PacBio-only assemblies**

Correction method	Starting PacBio coverage	% Assembly Size	Avg Identity	Indels < 5 bp	Indels > = 5	Inversions	Missing assembly contigs	Relocation	SNPs
Ion	110X	103.23%	99.98	859	3	0	0	1	103
Ion	185X	104.32%	99.98	893	4	0	0	1	94
MiSeq	110X	99.58%	99.99	149	3	3	0	3	117
MiSeq	185X	99.40%	99.99	66	2	1	0	2	104
Self-correction	110X	102.68%	99.96	1489	4	0	0	1	61
Self-correction	185X	103.48%	99.99	358	2	0	0	1	14

### PacBio-only assembly

Two recent software releases, from PacBio and the Celera Assembler group, allows for the error correction of PacBio reads without the need for short reads from another technology [16, 17]. To address the ability of these self-corrected reads to be assembled, we self-corrected both the 110× and 185× BL21(DE3) data sets using the PacBio software package. Prior to error-correction the average base Q score was ~ 9.6, post error-correction these qualities increased to between 47 and 57, indicating that error correction was successful. Similar to hybrid assembly, we downsampled based on length to coverages of approximately 12-15× and 20-25× (Additional file 3: Table S2). Each of the four data sets were assembled with ten different parameter sets using the Celera assembler. We were therefore left with 20 assemblies each for the original 185× PacBio data set and the 110× data set. The best for each of these coverages was again chosen using amosvalidate, scaffolded with AHA, and gap-filled with PBJelly. Remarkably, self-correction and assembly proved to be more complete than both the Ion Torrent and MiSeq-corrected assemblies across nearly every metric analyzed (Table 5).

The MUMmer/GAGE package showed that the PacBio-only assemblies were not only more complete than the hybrid-assemblies, but largely more accurate. Fewer SNPs, large indels, inversions and relocations were observed in the PacBio-only assembly as compared to the hybrid assemblies (Table 6). This accuracy improved as more PacBio data was added into the assembly, with only 14 SNPs in the final self-assembly, compared to 90 or more in each of the hybrid assemblies. Visual inspection of the dot-plots generated with the hybrid and PacBio-only assemblies also confirmed that PacBio-only assemblies were in fact more accurate than the hybrid-assemblies (Figure 4).

### Bal225 and DH5α assemblies

We next sought to sequence and assemble two additional *E. coli* strains for which reference genomes were not readily available. Given that the most complete assemblies were achieved with hybrid and PacBio-only assemblies, short read only assembly and hybrid scaffolding were rejected in favor of these methods. Again, over 100× coverage for each strain was generated on both the MiSeq and the Ion Torrent. PacBio long-insert libraries were prepared for both strains and sequenced to ~135× coverage for Bal225 (mean read length after adapter removal of ~ 2 kb) and ~200× coverage for DH5α (mean read length after adapter removal of ~2.4 kb).

As described above for the BL21(DE3) data, the PacBio long reads were corrected with either 100× MiSeq data or 100× Ion Torrent data using pacBioToCA, or self-corrected using PacBio’s Preassembler. For Bal225 we corrected all 135× coverage of PacBio long reads. DH5α data was randomly downsampled to ~100× coverage and both the full dataset (~200× coverage) and this subset were error corrected. For each data set, post-correction down-sampling was performed, twenty assemblies for each correction type-coverage combination were performed using the Celera assembler, and the best assemblies chosen.

Final assembly statistics are presented in Table 7. PacBio-only assemblies of Bal225 were again superior to both types of hybrid-assembly across nearly all metrics analyzed. Less than 20 contigs were assembled, with an N50 of over 1 MB, while the Ion Torrent hybrid assembly resulted in 31 contigs (N50 of 0.49 MB) and the MiSeq hybrid assembly with 54 contigs, and an N50 of only 0.13 MB. Hybrid assembly with Ion Torrent data proved to provide the most complete assembly with the DH5α strain, although again, the PacBio-only assemblies outperformed the MiSeq-hybrid assemblies by a considerable margin. Specifically, the PacBio-only assembly resulted in less than 20 scaffolds, while the MiSeq assemblies resulted in over 30 scaffolds.

**Table 7:** **Bal225 and DH5α assembly statistics**

Strain	Correction method	Starting PacBio coverage	Contigs	N50	Mean	Max	Total bases	Scaffolds	N50	Mean	Max	Total bases
Bal225	Ion	135X	31	489478	155895	1066404	4832765	26	2445003	185934	2445003	4834296
	MiSeq	135X	54	136035	84636	313301	4570378	33	265913	139590	436896	4606486
	Self-correction	135X	19	1024938	252532	2094681	4798125	16	1042373	300014	2094681	4800239
DH5alpha	Ion	95x	17	746888	276544	1350460	4701255	16	1016980	293829	1350460	4701268
		198X	6	2698624	769397	2698624	4616384	4	3138824	1154252	3138824	4617009
	MiSeq	95x	49	144080	92313	279069	4523338	31	279069	146814	664426	4551239
		198X	48	144514	93565	356157	4491145	35	189228	128837	694092	4509302
	Self-correction	95x	33	317772	140959	711007	4651647	19	503567	245691	1492059	4668131
		198X	35	274671	133071	845967	4657500	18	705151	259942	971908	4678967

### Assembly polishing

To reduce errors in the BL21(DE3) assembly, we next ran Quiver on each of the hybrid and PacBio-only assemblies [16]. Quiver is a software package that generates high quality consensus sequences by mapping long PacBio reads against a reference [16]. The assembled scaffolds were used as a reference, and uncorrected PacBio reads were used as the input to generate the consensus. After running Quiver on each assembly, we again evaluated the BL21(DE3) assemblies for correctness using the GAGE and MUMmer package described earlier. We found Quiver to be effective at reducing indels and SNPs, often dramatically improving the accuracy of the assembly (Table 8). In particular, for each of the BL21(DE3) assemblies, SNPs were reduced by 50% or more. Remarkably, the MiSeq hybrid assembly with 110x PacBio coverage went from 117 SNPs to 2. Small indels were also dramatically reduced, in some cases by more than 80%, demonstrating the utility of running Quiver as a final finishing step.

**Table 8:** **Improvements seen with Quiver**

Correction method	Starting PacBio coverage	Indels < 5 bp before Quiver	Indels < 5 bp after Quiver	Indels < 5 bp % improvement	Indels > = 5 before Quiver	Indels > = 5 after Quiver	Indels > = 5% improvement	SNPs before Quiver	SNPs after Quiver	SNPs % improvement
Ion	110X	859	292	66.01%	3	2	33.33%	103	30	70.87%
Ion	185X	893	173	80.63%	4	1	75.00%	94	10	89.36%
MiSeq	110X	149	73	51.01%	3	2	33.33%	117	2	98.29%
MiSeq	185X	66	56	15.15%	2	1	50.00%	104	28	73.08%
Self-correction	110X	1489	237	84.08%	4	2	50.00%	61	36	40.98%
Self-correction	185X	358	90	74.86%	2	1	50.00%	14	5	64.29%

### Base modifications

A unique feature of data generated with the PacBio is the ability to call base modifications. Identification of these modifications is based on the kinetics of base incorporation. When the interpulse distance ratio of base incorporation differs from expected, it indicates the presence of a modified base [39]. The specific kinetic signatures for 5mC, 6 mA, and 4mC can be reliably modeled and identified from sequencing data. To call 5mC base modifications, a specialized library preparation is required that increases the intensity signal above background [40]. Current protocols for this library preparation require 500 bp - 1 kb insert libraries treated with tetracycline. These libraries were generated for all three strains, and sequenced to coverages of at least 60× for each strain.

The *E. coli* strains used in this study were chosen specifically for their known methylase genotypes. The DH5α strain of *E. coli* has functional copies of all three methylases, and therefore all types of methylation should be detectable. As an *E. coli* strain B bacterium, BL21(DE3) naturally lacks the DCM methylase, and therefore we would expect not to find the methylated sequence motifs CCAGG and CCTGG. BL21(DE3) also lacks the HsdS subunit of EcoKI, required for sequence recognition, and we therefore do not expect these motifs to be methylated [41]. Finally, Bal225 is known to lack both the DAM and DCM methylases, and therefore we would expect not to find the motifs associated with these methylases to be methylated.

**Figure 5:**
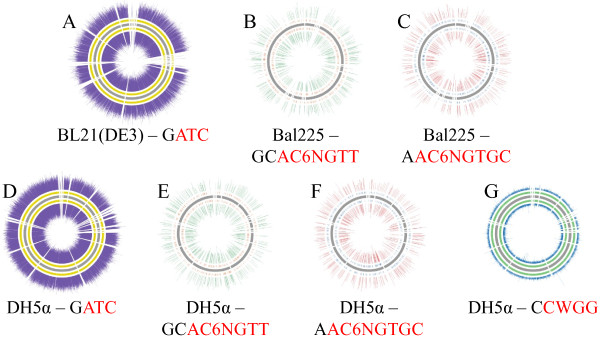
**Circos plots of base modifications.** The PacBio-only assembly was fed into SMRTAnalysis 1.4 and base modifications were called. In each figure, the assembled contigs are plotted as the inner grey bars. On either side of these grey contigs, the short lines indicate motif positions in the genome (the plus sense and minus sense are plotted). Outside of those are the location of the modifications and the intensity of those modifications. **A**. BL21(DE3) – yellow bars are positions of GATC motifs, purple are those motifs that are modified. **B**. Bal225, GCAC6NGTT. **C**. Bal225, AAC6NGTGC. **D**. DH5alpha, GATC. **E**. DH5alpha, GCAC6NGTT. **F**. DH5alpha, AAC6NGTGC. **G**. DH5alpha, CCWGG.

After sequencing the libraries to coverages of 60× or greater, we used SMRTAnalysis 1.4 to identify base modifications and enriched motifs. This module requires a reference sequence, and for these studies we used the scaffolds that resulted from self-correction and assembly performed earlier. The sequence motifs associated with all three methyltransferases were enriched in the DH5α samples. Specifically, over 98% of the motifs associated with the DAM methylase and EcoKI methylases were found to be modified. Detection of 5mC associated with the DCM methylase was not as strong, but significantly over background, with 40% of the motifs identified as methylated. As expected, in the Bal225 samples there was no enrichment for the DCM-related sequence motifs or the GATC motif, associated with the DAM methylase. The GATC motif in the BL21(DE3) samples were enriched, with approximately 97% of the motifs identified as methylated, while lacking all other modifications, as expected. In short, all expected modifications were identified, with no false positives. Table 9 shows the methylation patterns identified for each strain, and Figure 5 shows the location of each motif and each modified motif mapped against the assembled scaffolds.

**Table 9:** **Identified methylation patterns**

Strain	Genotype	6 mA - Enriched motifs(percent)			5mC
BL21(DE3)	dam(+), dcm(−), hsdSB(rB- mB-)	G**A**TC (94.83)			None
Bal225	dam(−), dcm(−)	A**A**C6NGTGC (97.86)	GC**A**C6NGTT (96.71)		None
DH5α	dam(+), dcm(+)	A**A**C6NGTGC (98.15)	GC**A**C6NGTT (98.82)	G**A**TC (98.49)	C**C**WGG (40.20)

The nucleotide bases in bold are the nucleotides that are covalently modified by their respective enzymes.

## Discussion

In this study we explore a variety of methodologies for the *de novo* assembly of bacterial genomes and analyze the epigenetic base modifications associated with the *E.coli* strains, BL21(DE3), DH5α, and Bal225. Understanding how best to assemble bacterial genomes *de novo* is important for at least two reasons. First, bacteria play an important role in nearly all ecological and biological processes on Earth. Full knowledge of how these bacteria interact with the world around them requires an understanding of their underlying genetic architecture. Second, bacterial genomes are relatively simple when compared to more complex eukaryotic genomes. Thus, a firm understanding of how best to assemble bacterial genomes can inform the assembly of larger, more complex genomes.

Here we examined four different methodologies for the assembly of bacterial genomes: short read only assembly, hybrid scaffolding, hybrid assembly, and PacBio-only assembly (Figure 1). To evaluate the effectiveness of each strategy we generated 100× coverage of the three strains on both the Life Technologies Ion Torrent and the Illumina MiSeq and at least 125× coverage with long-insert reads on the PacBio RS for all three strains.

As was expected, the assemblies with the greatest number of contigs came from assemblies using either the Ion Torrent or MiSeq data alone (short read only). For these studies we examined three commonly used assemblers, Velvet, Ray, and MIRA [29–31]. Both Velvet and Ray are de Bruijn graph-based assemblers. These assemblers are known to be less tolerant of sequencing errors, which may explain why they struggled with the Ion Torrent data whose Q scores were slightly below that of the MiSeq data (28.8 vs. 34.6, Additional file 1: Table S1) [42]. We performed a kmer and coverage parameter sweep with Velvet and the MiSeq data, examining 48 different assemblies. Velvet was capable of assembling the MiSeq data effectively, with generally less than 200 contigs that were typically longer than any of the other short read assemblies. MIRA, which is not a de Bruijn graph assembler, was able to assemble both sets of data, producing the lowest number of contigs with Ion Torrent data, although of the three methods, MIRA had the most trouble with the MiSeq data. Ray stood apart as the most accurate of the three assemblers, based on the number of inversions, relocations, SNPs, and a visual inspection of the associated dot plots (Table 2, Figure 2). These more accurate assemblies did not come at a cost of assembly completeness (Table 1). In particular, the Ray-MiSeq assemblies were often the most complete, with contigs of 100 or less for three of the coverages, the only short read assembler-data combination to achieve such results.

One interesting finding from this study is that more short read coverage does not necessarily guarantee a better assembly. We found that lower coverages, especially for the Ion Torrent data, often resulted in assemblies that were similar to those generated with higher coverage. This is not entirely unexpected for the MIRA assemblies, as overlap graph based assemblers are less tolerant of high coverage [42]. However this observation held true for the Ray-Ion Torrent assemblies as well. We should also note here that while it is typically thought that paired-end data is significantly better for assembly than is single-end data, there was little difference in assembly completeness between the best MiSeq assembly (performed with Ray) and the best Ion Torrent assembly (assembled with MIRA).

After generating these short read assemblies, we chose 1 representative assembly from each data:assembler combination (highlighted in Table 1) and attempted to connect the contigs with long, uncorrected PacBio reads. Hybrid scaffolding resulted in significant assembly improvements for all scenarios examined, with an average of 50% reduction in contig number across all PacBio coverages and an average of 5-fold improvements in N50 values. Although much of the gains were realized with just 25× PacBio coverage, improvements did increase incrementally as more PacBio reads were added to the assembly. The assembly that seemed to benefit the most from hybrid scaffolding was the Ray-Ion Torrent assembly (Table 3). This is not terribly surprising, as the Ray-Ion Torrent assemblies were the most accurate, and yet the most fractured, and therefore should be the easiest to connect. When 110× PacBio coverage was used in hybrid scaffolding, contigs were reduced by 80% and the N50 length went up by more than 10-fold. What was surprising was the number of errors introduced by using AHA/PBJelly. Far more relocations, inversions, indels, and SNPs are present in these assemblies than in the short read only assemblies (Table 4 and Figure 3). Errors in hybrid scaffolding represent overly aggressive attempts to connect contigs, some of which are connected erroneously. Others have shown it possible to effectively use PacBio data for scaffolding when implemented as a part of the ALL-PATHS LG sequence assembler recipe [43]. Therefore, it should be possible to reduce the aggressiveness of this process in order to eliminate some of these introduced errors, and others may be resolved by running Quiver post-assembly. Reducing the aggressiveness of contig scaffolding will result in less complete assemblies, but the gains made in accuracy may be acceptable in some circumstances. In spite of these potential errors, we employed the hybrid scaffolding technique on all subsequent assemblies. Often, the goal of assemblies is to achieve as complete an assembly as possible. There are always tradeoffs to be made, but in the end we believed that the gains resulting from scaffolding were worth the potential of introduced errors.

While short read only assemblies are still popular because of the relative newness, cost of entry, and throughput concerns associated with long read sequencing technology, the state of the art in genome assembly lies with the long reads generated by the PacBio. We therefore wanted to see how hybrid assembly and PacBio-only assemblies would compare with short read only assemblies and each other. Unexpectedly, the Ion Torrent error-corrected reads assembled far more efficiently for each of the three strains examined across all coverages and parameter sweeps when compared to MiSeq error-corrected reads. These results can be traced back to longer corrected reads post-Ion Torrent correction. This may be due to the fact that the Ion Torrent reads themselves are longer than the MiSeq reads. These longer reads should be easier to map back to the PacBio reads, increasing error correction efficiency. It’s also possible that differences may be due to the manner in which the MiSeq libraries were generated. MiSeq libraries were made using the Nextera kit, which fractures DNA with transposons as opposed to the mechanical shearing used to create the PacBio and Ion Torrent libraries or chemical shearing typical of other Illumina library preparation kits. The insert sizes associated with these libraries were far more varied than what is typically encountered with Illumina libraries, and this may have contributed to the poorer performance of the MiSeq data in both hybrid assembly and the short read only data assembly using MIRA (Additional file 2: Figure S1).

We used Preassembler with the same PacBio data that was used in the previous analyses. Remarkably, the PacBio-only assemblies were superior to the MiSeq-PacBio hybrid assembly across all strains and coverages examined. Furthermore, the completeness of these assemblies were generally comparable to, and often slightly superior to the best Ion Torrent-PacBio hybrid assemblies (Tables 5 and 7). Perhaps even more impressively, the BL21(DE3) PacBio-only assembly was the most accurate of all three types (Table 6). This accuracy improves even further when one finishes the assembly with Quiver (Table 8).

The assembly results here fall largely in line with two recent papers [16, 17]. Chin *et al.* was the first paper to demonstrate the effectiveness of both self-correction using the PacBio software package and Quiver. In Koren, *et al.* investigators describe using the Celera assembler package to self-correct PacBio long reads, as well as perform hybrid and short read assemblies. Similar to the results shown here, the investigators found that self-correction of PacBio long reads lead to as good, or better, assemblies than hybrid-based approaches, and that assembly polishing with the Quiver package led to highly accurate assemblies [17]. This investigation diverges slightly from these two reports in that we unable to close the genome of the three investigated *E. coli* strains. Closing the genome of microbes is generally thought to be highly correlated to the number and size of the repetitive elements found in the sequenced genomes. Sequence reads must span the repeat regions in order to properly resolve these elements. When these reads are not present, gaps will occur. Two factors are thus important when considering whether or a bacterial genome can be closed – the expected maximum length of repetitive elements in the genome of interest, and the length of the sequencing reads. Read lengths of the corrected reads must be longer than the longest repeat, and have sufficient depth as to cover and resolve the repeat regions. For BL21(DE3), post-error correction reads averaged 5000 nt and 5500 nt with ~20X coverage. This should be close to the necessary lengths needed to resolve these repetitive elements, but were not sufficient in this case.

It should be noted that in the time since this data has been generated, both Illumina and Life Technologies have introduced sequencing kits that produce even longer reads than what was used here – both platforms yield sequence reads that are twice as long as what was used in this study. These reads will undoubtedly improve assemblies with data generated solely by these machines. Additionally, hybrid assemblies should be improved, as longer short read data seems to result in longer error-corrected reads. Still, given the difficulty these two technologies have with repetitive sequences and read lengths that still fall far short of those produced by PacBio, it is unlikely that these advancements would alter any of the conclusions made here. However, Illumina has recently purchased a technology that rivals the PacBio in read length, known as Moleculo sequencing. This technology stitches together standard Illumina reads into long reads of approximately 10 kb in length. These reads have the advantage of being both high quality and long, eliminating the need for error correction. Unfortunately since it is based on stitching together short reads, resolution of repetitive regions is likely to remain difficult.

Until Moleculo becomes widely available, and the question of repetitive sequence resolution can be answered, the PacBio should be the platform of choice for any *de novo* bacterial assembly. In addition to superior assemblies, the PacBio offers a unique capability – the ability to call covalent base modifications. Currently PacBio software can detect three types of base modifications, 6 mA, 5mC, and 4mC. The three strains in this study were specifically chosen to test the specificity and sensitivity of the PacBio sequencer and associated software to call modifications. The BL21(DE3) is a type B strain of *E.*coli, naturally lacking the DCM methylase, and therefore we expected to see no modifications of the CC×GG motif in this strain and did not. We failed to find enrichments of the motifs associated with EcoKI, but in contrast, high rates of GATC modification were both expected and found. Bal225 was expected to be both DAM and DCM deficient, and while nearly every EcoKI motif was found to be modified, no enrichments for the GATC or CC×GG motifs were detected. Finally DH5α served as a positive control, as it is known to be wild-type for all three methyltransferases. Indeed, we found all three motifs to be modified. For this strain, those motifs associated with 6 mA were highly modified, while the 5mC motifs CC×GG were detected as modified ~40% of the time. 5mC motifs are more difficult for the PacBio software to detect, and a special library preparation is required to call these modifications [40]. We cannot rule out the possibility that this library preparation (which includes the treatment of DNA with tetracycline) was not 100% effective in marking all modified bases, however, a recent report indicates that not all CC×GG motifs are modified, and this may explain the lower level of methylation found here [44].

## Conclusions

In summary, we compare and contrast competing methods for the assembly of bacterial genomes, demonstrating that PacBio-only assembly is comparable to hybrid assembly and significantly superior to assemblies performed with short read only data. We go on to demonstrate the sensitivity and specificity of calling base modifications using PacBio data.

Moving forward, the results presented here demonstrate that to obtain the most complete and accurate assembly of a bacterial-sized genome, researchers should generate at least 100× coverage data on the PacBio. This data should then be self-corrected using the PacBio SMRT Analysis software or the Celera error correction module, and assembled using Celera [14–17]. A recent report demonstrates that if enough long read data is obtained, a single contig will be the end result, however if individual contigs remain, researchers can improve the assembly by scaffolding with AHA, and gap-filling with PBJelly [15–17, 19]. Finally, using Quiver as a final error correction step will improve the accuracy of the assembly even further and should be implemented to ensure the most accurate assembly possible.

## Methods

### E.coli strains

*E. coli* strains BL21(DE3), Bal225, and DH5α™(Life Technologies, Inc. Grand Island, NY USA) were grown in LB broth to concentrations of approximately 1×10^9^ cells/ml, and genomic DNA was isolated with Qiagen® DNeasy® Blood and Tissue Kit (Qiagen, Inc. Germantown, MD USA).

### Life technologies ion torrent library preparation and sequencing

Genomic DNA sequencing was conducted using the Life Technologies Ion Torrent Personal Genome Machine™ (PGM™). Libraries were made using the Ion Plus Fragment Library Kit (Life Technologies Item # 4471252). Briefly, purified genomic DNA was fragmented to a size range of approximately 200–300 bp using the Covaris® E210 instrument (Covaris Inc. Woburn, MA USA). Fragmented DNA was repaired and made blunt ended, then purified using Ampure XP® Beads (Beckman Coulter Inc. Atlanta, GA USA; Item# A63880). Ion Sequencing adapters and Ion Express™ barcodes (Life Technologies Item # 4474518) were then ligated to the blunt-ended DNA fragments, purified using Ampure XP® Beads, then size-selected to 330 bp using an E-Gel® SizeSelect™ Agarose Gel (Life Technologies Item # G661002). The size selected product was amplified by PCR, then purified using Ampure XP® Beads. The resulting DNA library was quantified using the Agilent Bioanalyzer DNA Chip (Agilent Technologies, Inc. Santa Clara, CA USA; Item # 5067–4626). The libraries were pooled at equimolar concentrations and clonally amplified and enriched onto Ion Spheres using the Ion One Touch™ Template Prep System and the Ion Torrent One Touch™ PGM 200 Kit (Life Technologies Item # 4478316). Enriched Templated Ion Spheres were deposited onto a semiconductor chip (Ion 318™ Chip, Life Technologies Item # 4466617) and sequenced using the PGM™ Instrument and the Ion PGM™ 200 Seq Kit (Life Technologies Item # 4474004). Ion Torrent Suite software (version 3.2.1) was used to convert raw signal to Base Calls and generate FASTQ files for subsequent analysis.

### Illumina MiSeq library preparation and sequencing

Bacterial genomic DNA was prepared for sequencing on the Illumina MiSeq using the Nextera DNA Sample Prep Kit (Illumina, Inc. San Diego, CA USA; Item # FC-121-1030). Steps were performed as described in the Nextera DNA Sample Preparation Guide (Item # 15027987 Rev. B October 2012). Briefly, genomic DNA was tagmented (tagged with PCR adapters and fragmented), followed by purification of tagmented DNA and limited-cycle PCR (during which indexes, sequencing adapters, and common adapters are added for subsequent cluster generation and sequencing). PCR library DNA was then purified using Agencourt AMPure XP beads (Beckman Coulter, Item # A63882), which excluded very short library fragments. DNA libraries were then quantified using the Qubit assay and qualified using the Agilent Technologies High Sensitivity DNA Kit (Item # 5067–4626). Purified libraries were pooled and sequenced on the MiSeq using a 2×150 paired-end protocol. Initial basecalls were converted to fastq files using MiSeq CASAVA software suite [45].

### Pacific Biosciences RS library preparation and sequencing

Three libraries were prepared for each strain: long insert, long insert with Tet1-treatment and 1 kb insert with Tet1-treatment. Genomic DNA samples were sheared to target insert size (10 kb or 1 kb) depending on the chosen sequencing strategy using a Covaris® Adaptive Focused Acoustics instrument, or the g-Tube, also from the Covaris®. Fragmented DNA was then purified using AMPure® PB magnetic beads and verified on an Agilent Bioanalyzer DNA Chip. Tet1-treatment was carried out on intended fragmented DNA according to guidelines for using the WiseGene™ 5-mC Tet1 oxidation kit for SMRT® sequencing on the Pacific Biosciences® RS (WiseGene LLC., Chicago, IL USA; Item #K004; Pacific Biosciences Inc. Menlo Park, CA USA). Libraries were subsequently prepared following PacBio guidelines. End-repair was performed, followed by ligation of universal hairpin adapters to produce the SMRTbell library. SMRTBell libraries were verified using Life Technologies Qubit® 2.0 and the Agilent Bioanalyzer. The PacBio specific sequencing primer was annealed to the SMRTbell library followed by binding of the polymerase to the primer-library complex. Libraries were loaded onto the SMRT cells with the assistance of the MagBead stations and sequenced on the PacBio RS system. Long-insert libraries were sequenced with stage-start settings, and used 1×120 movies, while the short libraries were sequenced with 2×55 movies. Both libraries were sequenced using the C2 chemistry and C2-XL enzyme.

### Pre-processing

Ion Torrent data was de-multiplexed using Ion Torrent Suite software (version 3.2.1). MiSeq data was de-multiplexed using internally developed software package fastq-multx (available for free download from https://code.google.com/p/ea-utils/) [34]. Unless otherwise noted, all data was clipped for adapters and quality scores with fastq-mcf, also internally developed and available for download [46]. PacBio reads were processed with the SMRT Analysis module RS_Filter_and_Control_Pmodules which removes SMRT bell adapters and spike-in sequences.

### Short read assemblies

Using randomFQ BL21(DE3) reads from the MiSeq and Ion Torrent were downsampled to approximate coverages of 25×, 50×, 75×, and 100×. MiSeq reads were assembled with the Velvet (v. 1.2.08) assembly software package [29] using default parameters, but with varying kmer lengths. Specifically, kmers were varied from 21 to 63. Statistics such as contig number, N50, and max contig length were generated from each assembly using the script contig-stats (available for download from ea-utils), and visually inspected. Based on these statistics, a kmer of 59 was chosen as consistently among the best for the four coverages examined. In a similar manner, assemblies with the Ray assembler (v. 2.1.0) [30] were performed with default settings, again with varying kmer lengths (ranging from 21 to 61). Ray was capable of assembling both the Ion Torrent and MiSeq data. Assemblies were again inspected for completeness, and based on these statistics, a kmer of 36 for the MiSeq data and 29 for the Ion Torrent data consistently resulted in the best assemblies across the four different coverages. Finally, both the Ion Torrent and MiSeq data were assembled using MIRA (v. 3.9.9) [31]. For the Ion Torrent data, we set the technology to “iontor” and used the default parameters for analysis. We chose not to clip the MiSeq data before loading into MIRA, opting instead to allow MIRA’s internal clipping algorithm to perform this step by setting CL:pvlc = on:qc = on and the technology to “solexa”.

### Hybrid-error correction

PacBio long reads were error-corrected by 100x coverage of either MiSeq reads or Ion Torrent reads essentially as described [12]. Briefly, MiSeq and Ion Torrent fastq files were converted to pacBioToCA compatible frg files with fastqToCA, also part of the Celera assembler package [14]. These frg files were then used as input, along with the uncorrected reads into pacBioToCA. Post-error correction reads were downsampled based on length to two coverages, between 12 and 25× using internally developed scripts [34].

### Preassembler

For each strain the associated SMRT cells were loaded into the SMRT Analysis package [15]. SMRT cells were chosen in the interface for use in each set of corrections. Minimum Seed Read Length was chosen to get two coverages between 12 and 25×. The BLASR “–maxLCPLength” option was set to 14, and “Trim FASTQ Output” was turned off [47]. Post-correction we ran trimFastqByQVWindow.py on the corrected fastqs with cutoffs of 19 or 49, ultimately choosing the cutoff that placed the data in the desired coverage range.

### Celera assembly

Celera assembler (v. 7.0) [14] was used to assemble the corrected reads. Ten different parameter settings for each data set was used, mostly variations of ErrorRates and merSize (Additional file 4: Table S3).

### Assembly assessment

BL21(DE3) assemblies were compared to the NCBI reference using the GAGE script which interfaces with MUMmer [35, 36]. Celera assemblies were assessed with amosvalidate and FRCurve as described [37, 38].

### Scaffolding

After assembly, contigs were imported into the SMRT Analysis package and used as the reference for A Hybrid Assembler (AHA). PacBio reads were used to scaffold the contigs using default parameters. The resultant scaffolds were gap-filled with PBJelly [19], again using default parameters.

### Covalent base modifications

Base modifications were found using the SMRT Analysis package and the accompanying package RS Modification and Motif Analysis. Circos plots were generated with internally developed scripts and the Circos graphics package [48].

### Availability of supporting data

The data sets supporting the results of this article are available in the SRA repository, (Bal225: PRJNA203022; BL21(DE3): PRJNA203015). Custom scripts used to analyze the data are available on the ea-utils FASTQ processing utilities website https://code.google.com/p/ea-utils/.

## Electronic supplementary material

Additional file 1: Table S1: BL21(DE3) sequencing statistics. (PDF 32 KB)

Additional file 2; Figure S1: Insert size distribution of MiSeq reads. BL21(DE3) MiSeq reads were aligned to the BL21(DE3) reference and the calculated insert sizes were plotted using R and ggplot2 (in navy blue). Previous data generated with an Illumina TruSeq kit and the *E. coli* strain DH10b was similarly mapped to its reference and insert sizes plotted (in red). Note the bimodal distribution of the BL21(DE3) reads. (PPTX 86 KB)

Additional file 3: Table S2: Post-error correction statistics. (PDF 31 KB)

Additional file 4: Table S3: Celera spec file parameters. (PDF 36 KB)

Additional file 5: Figure S2: An example FRCurve. This FRCurve was generated from the BL21(DE3) Ion Torrent hybrid assembly with 185x PacBio coverage and the Celera assembler. Amosvalidate and FRCurve were used to analyze 20 different assemblies. Amosvalidate-identified features (representing potential mis-assemblies) were plotted on the x-axis with approximate genome coverage on the y-axis. The assembly with the lowest number of features at 95% genome coverage was identified as the best assembly. Here, that assembly is highlighted in black, and corresponds to Celera spec file parameters “Run 1” in Additional file 4: Table S3. (PPTX 86 KB)

## Abbreviations

PacBio: Pacific Biosciences
RS: AHA, A Hybrid Scaffolder
DAM: DNA adenine methylase
DCM: DNA cytosine methylase.
