# Supplementary material for: Efficient and accurate whole genome assembly and methylome profiling of E. coli
Source: BMC Genomics. 2013 Oct 3;14(1):675. doi: 10.1186/1471-2164-14-675 (PMC4046830; doi:10.1186/1471-2164-14-675)
Supplement: Supplementary file 1 — Additional file 1: Table S1: BL21(DE3) sequencing statistics. (PDF 32 KB) [file 12864_2013_5438_MOESM1_ESM.pdf]

Table S1 – BL21(DE3) sequencing statistics

Machine	Type of Sequencing	Avg Read Length	Avg Quality	Total Bases
Ion Torrent	Single-end, 318 chip	199	28.84	4.93E+08
Illumina MiSeq	Paired-end 150bp, Nextera Kit	131	34.56	4.97E+08
PacBio RS	Long-insert, 1x120 movies	1909	9.73	8.39E+08
