# Supplementary figures and images for: Efficient and accurate whole genome assembly and methylome profiling of E. coli
Source: BMC Genomics. 2013 Oct 3;14(1):675. doi: 10.1186/1471-2164-14-675 (PMC4046830; doi:10.1186/1471-2164-14-675)

## Slide 1
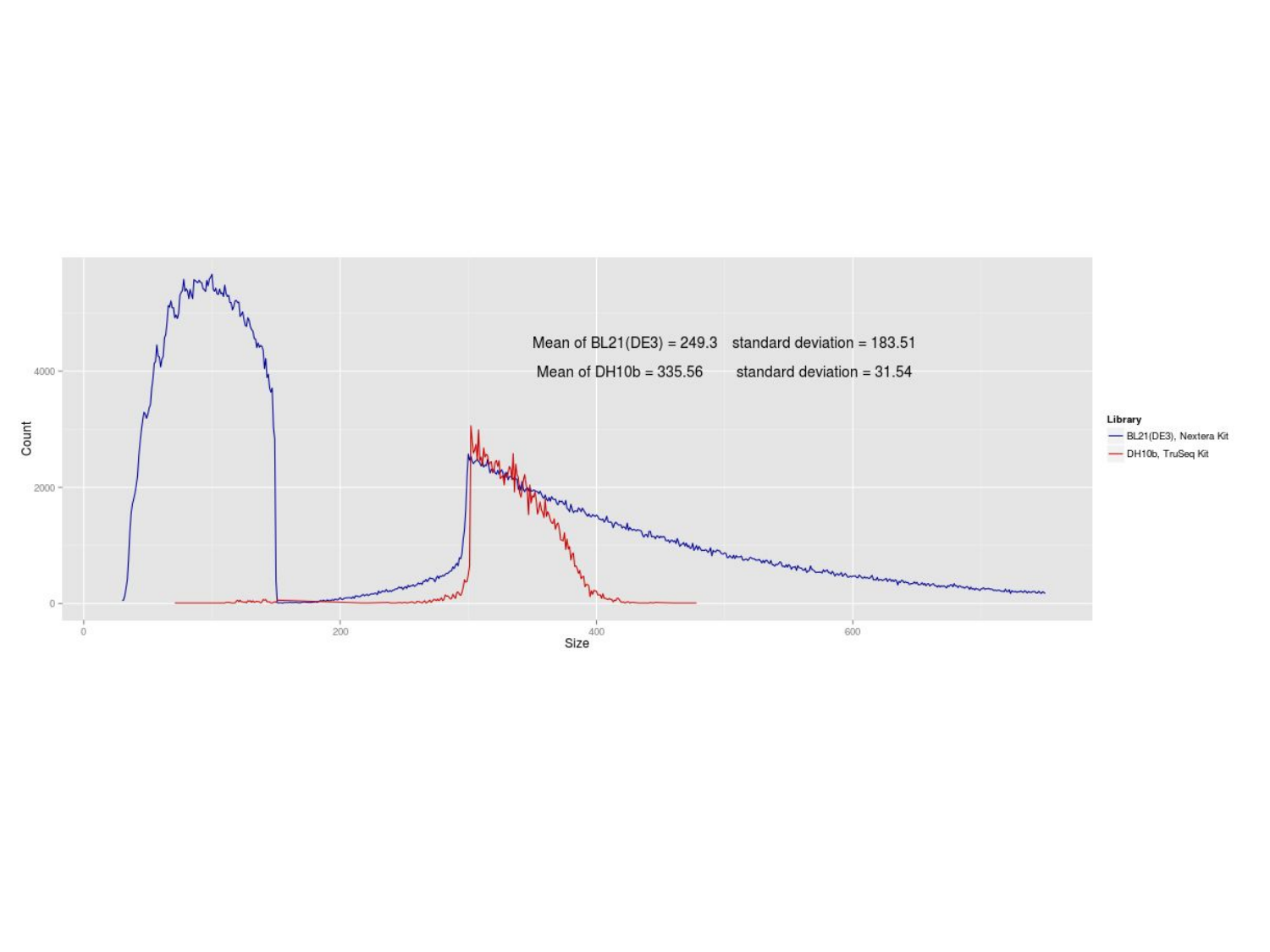

Supplement: Supplementary file 2 — Additional file 2; Figure S1: Insert size distribution of MiSeq reads. BL21(DE3) MiSeq reads were aligned to the BL21(DE3) reference and the calculated insert sizes were plotted using R and ggplot2 (in navy blue). Previous data generated with an Illumina TruSeq kit and the E. coli strain DH10b was similarly mapped to its reference and insert sizes plotted (in red). Note the bimodal distribution of the BL21(DE3) reads. (PPTX 86 KB) [file 12864_2013_5438_MOESM2_ESM.pptx]

## Slide 1
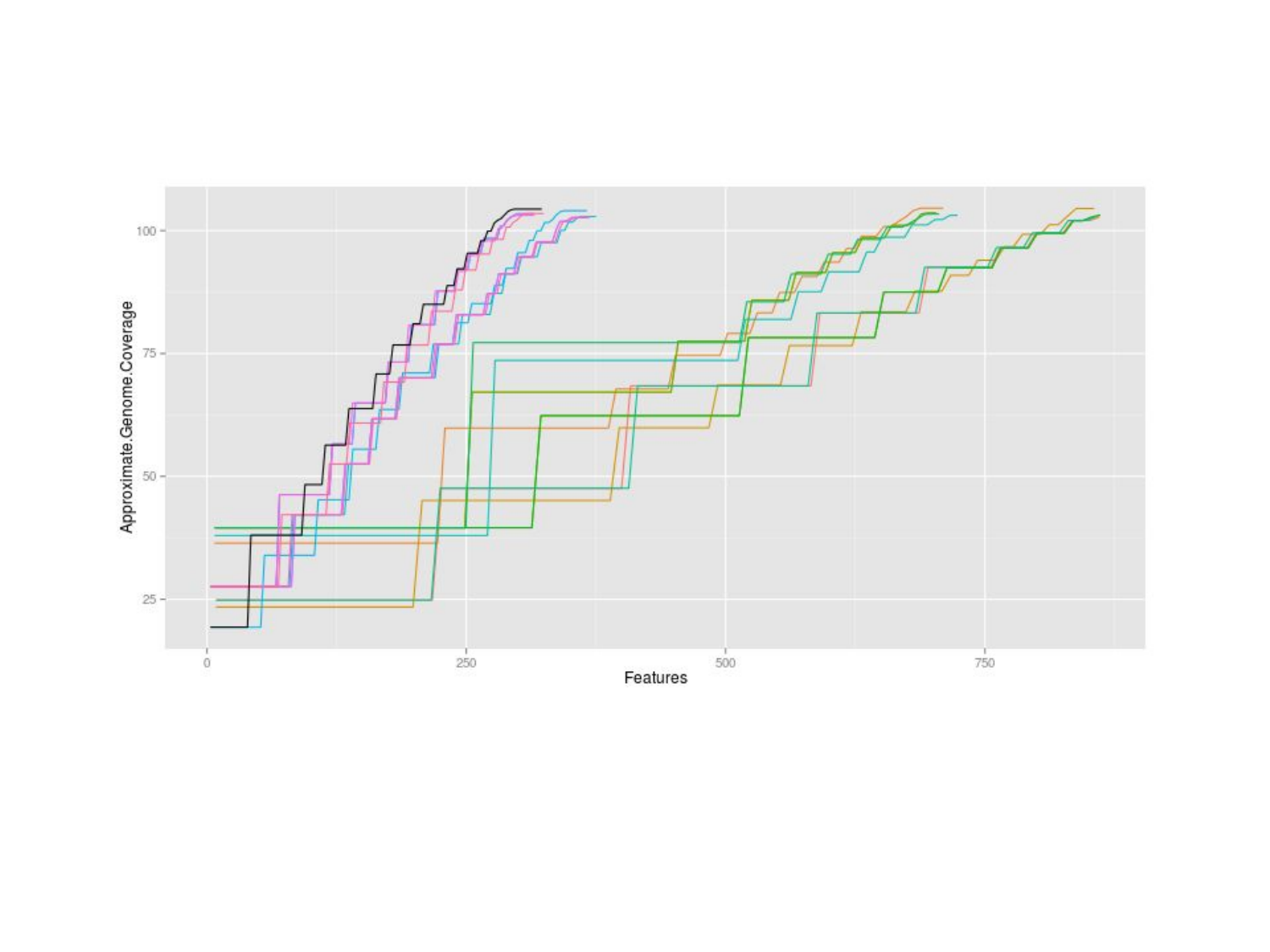

Supplement: Supplementary file 5 — Additional file 5: Figure S2: An example FRCurve. This FRCurve was generated from the BL21(DE3) Ion Torrent hybrid assembly with 185x PacBio coverage and the Celera assembler. Amosvalidate and FRCurve were used to analyze 20 different assemblies. Amosvalidate-identified features (representing potential mis-assemblies) were plotted on the x-axis with approximate genome coverage on the y-axis. The assembly with the lowest number of features at 95% genome coverage was identified as the best assembly. Here, that assembly is highlighted in black, and corresponds to Celera spec file parameters “Run 1” in Additional file 4: Table S3. (PPTX 86 KB) [file 12864_2013_5438_MOESM5_ESM.pptx]
