# Supplementary material for: Efficient and accurate whole genome assembly and methylome profiling of E. coli
Source: BMC Genomics. 2013 Oct 3;14(1):675. doi: 10.1186/1471-2164-14-675 (PMC4046830; doi:10.1186/1471-2164-14-675)
Supplement: Supplementary file 3 — Additional file 3: Table S2: Post-error correction statistics. (PDF 31 KB) [file 12864_2013_5438_MOESM3_ESM.pdf]

Table S2 – Post-error correction statistics

Correction Method	Approx. PacBio coverage	Avg. length pre-correction	Avg. Qscore pre-correction	Approx. coverage post-correction	Yield	Avg. length post-correction	Avg. Qscore post-correction	20-25X coverage	20-25X avg. length	20-25X avg Qscore	10-15X coverage	10-15X avg. length	10-15X avg. Qscore
Ion	112	1998.79	9.69	81.04	0.72	1556.35	55.85	22.62	4655.82	56.25	15.03	5661.46	56.34
	186	1909.00	9.73	136.30	0.73	1564.18	55.98	22.65	5515.19	56.44	13.84	6510.52	56.50
MiSeq	112	1998.78	9.69	72.8	0.65	1403.43	57.39	20.9	3925.38	57.49	14.5	4725.64	57.53
	186	1908.99	9.73	124.5	0.67	1429.89	57.47	23.8	4627.20	57.62	14	5620.57	57.64
Preassembler	112	1998.79	9.69					22.72	5094.44	46.29	15.41	5500.94	57.75
	186	1909.00	9.73					23.04	5512.50	57.93	16.28	6335.60	58.18
