# Supplementary material for: Efficient and accurate whole genome assembly and methylome profiling of E. coli
Source: BMC Genomics. 2013 Oct 3;14(1):675. doi: 10.1186/1471-2164-14-675 (PMC4046830; doi:10.1186/1471-2164-14-675)
Supplement: Supplementary file 4 — Additional file 4: Table S3: Celera spec file parameters. (PDF 36 KB) [file 12864_2013_5438_MOESM4_ESM.pdf]

Table S3 – Celera spec file parameters

	Run 1	Run 2	Run 3	Run 4	Run 5	Run 6	Run 7	Run 8	Run 9	Run 10
**ovlErrorRate**	0.2	0.06	0.06	0.1	0.1	0.1	0.1	0.1	0.1	0.2
**cnsErrorRate**	0.2	0.06	0.06	0.1	0.1	0.1	0.1	0.1	0.1	0.2
**cgwErrorRate**	0.2	0.1	0.1	0.1	0.1	0.2	0.2	0.2	0.2	0.2
**merSize**	11	22	11	22	11	22	11	22	11	22
**utgGraphErrorRate**	0.1	0.03	0.03	0.07	0.07	0.07	0.07	0.1	0.1	0.1
**utgGraphErrorLimit**	3.25	3.25	3.25	3.25	3.25	3.25	3.25	3.25	3.25	3.25
**utgMergeErrorRate**	0.045	0.045	0.045	0.045	0.045	0.045	0.045	0.045	0.045	0.045
**utgMergeErrorLimit**	5.25	5.25	5.25	5.25	5.25	5.25	5.25	5.25	5.25	5.25
**frgMinLen**	40	40	40	40	40	40	40	40	40	40
**ovlMinLen**	20	20	20	20	20	20	20	20	20	20
**doToggle**	0	0	0	0	0	0	0	0	0	0
**utgBubblePopping**	1	1	1	1	1	1	1	1	1	1
**doOverlapBased Trimming**	1	1	1	1	1	1	1	1	1	1
**doDeDuplication**	0	0	0	0	0	0	0	0	0	0
**unitigger**	bogart	bogart	bogart	bogart	bogart	bogart	bogart	bogart	bogart	bogart
**cleanup**	light	light	light	light	light	light	light	light	light	light
**cnsConcurrency**	16	16	16	16	16	16	16	16	16	16
**frgCorrBatchSize**	100000	100000	100000	100000	100000	100000	100000	100000	100000	100000
**frgCorrConcurrency**	8	8	8	8	8	8	8	8	8	8
**frgCorrThreads**	4	4	4	4	4	4	4	4	4	4
**merylMemory**	128000	128000	128000	128000	128000	128000	128000	128000	128000	128000
**merylThreads**	4	4	4	4	4	4	4	4	4	4
**ovlConcurrency**	6	6	6	6	6	6	6	6	6	6
**ovlCorrBatchSize**	100000	100000	100000	100000	100000	100000	100000	100000	100000	100000
**ovlCorrConcurrency**	16	16	16	16	16	16	16	16	16	16
**ovlCorrOnGrid**	0	0	0	0	0	0	0	0	0	0
**ovlHashBits**	24	24	24	24	24	24	24	24	24	24
**ovlHashBlockLength**	20000000	20000000	20000000	20000000	20000000	20000000	20000000	20000000	20000000	20000000
**ovlRefBlockSize**	5000000	5000000	5000000	5000000	5000000	5000000	5000000	5000000	5000000	5000000
**ovlStoreMemory**	8192	8192	8192	8192	8192	8192	8192	8192	8192	8192
**ovlThreads**	4	4	4	4	4	4	4	4	4	4
